# Investigating the impact of overnight fasting on intrinsic functional connectivity: a double-blind fMRI study

**DOI:** 10.1007/s11682-017-9777-9

**Published:** 2017-10-25

**Authors:** Stelios Orfanos, Timur Toygar, Mark Berthold-Losleben, Natalya Chechko, Annette Durst, Zacharias G. Laoutidis, Sebastian Vocke, Caren Weidenfeld, Frank Schneider, Wolfram Karges, Christian F. Beckmann, Ute Habel, Nils Kohn

**Affiliations:** 1 Department of Psychiatry, Psychotherapy and Psychosomatics, Medical Faculty, RWTH Aachen University, Aachen, Germany; 2 Jülich Aachen Research Alliance (JARA) - BRAIN Institute Brain Structure-Function Relationships: Decoding the Human Brain at systemic levels, Forschungszentrum Jülich GmbH and RWTH Aachen University, Jülich, Germany; 3 Department of Biology, RWTH Aachen University, 52074 Aachen, Germany; 4 Department of Psychiatry and Psychotherapy, University of Düsseldorf, Bergische Landstrasse 2, 40629 Düsseldorf, Germany; 5 Division of Endocrinology and Diabetes, Medical Faculty, RWTH Aachen University, 52074 Aachen, Germany; 6 Department of Cognitive Neuroscience, Radboud University Medical Center, Donders Institute for Brain, Cognition and Behaviour, Nijmegen, The Netherlands; 7 Centre for Functional MRI of the Brain (FMRIB), University of Oxford, Oxford, UK

**Keywords:** Blood glucose, fMRI, Resting-state, ICA, Default mode, Strength model

## Abstract

The human brain depends mainly on glucose supply from circulating blood as an energy substrate for its metabolism. Most of the energy produced by glucose catabolism in the brain is used to support intrinsic communication purposes in the absence of goal-directed activity. This intrinsic brain function can be detected with fMRI as synchronized fluctuations of the BOLD signal forming functional networks. Here, we report results from a double-blind, placebo controlled, cross-over study addressing changes in intrinsic brain activity in the context of very low, yet physiological, blood glucose levels after overnight fasting. Comparison of four major resting state networks in a fasting state and a state of elevated blood glucose levels after glucagon infusion revealed altered patterns of functional connectivity only in a small region of the posterior default mode network, while the rest of the networks appeared unaffected. Furthermore, low blood glucose was associated with changes in the right frontoparietal network after cognitive effort. Our results suggest that fasting has only limited impact on intrinsic brain activity, while a detrimental impact on a network related to attention is only observable following cognitive effort, which is in line with ego depletion and its reliance on glucose.

## Introduction

The adult human brain depends mainly on glucose for its metabolism (Clarke and Sokoloff 1999) and although it is capable of utilizing alternative energy substrates, such as ketone bodies, it does so only in states of low glucose supply, e.g. in prolonged fasting or during starvation (Hasselbalch et al. 1994). As endogenous brain glucose reserves are minimal, the brain depends on blood flow for a continuous glucose supply in order to maintain its normal function (Hyder 2009). This permanent dependence becomes apparent in the context of hypoglycemia, where decreasing peripheral blood glucose levels (BGL) immediately lead to a series of events known as hypoglycemic symptoms. These range from feelings of anxiety and irritability to blurred vision and impairment of cognitive function (Mitrakou et al. 1991), while extreme low BGL can lead to coma and eventually brain death (de Courten-Myers et al. 2000). However, hypoglycemia constitutes a rare event in healthy humans as glucose levels are highly regulated through a series of neuronal and hormonal factors that maintain BGL between approximately 4,0 and 8,0 mmol/L (72–144 mg/dl) regardless of food intake or physical activity (Cryer 1997). Along with the cessation of insulin secretion, glucagon secretion is the primary glucoregulatory factor that prevents hypoglycemia (Cryer et al. 1984).

Exhibiting a high energetic demand, the adult human brain accounts for approximately 25% of the body’s total glucose metabolism, while consisting only 2% of the body’s total weight (Magistretti and Allaman 2013). Approximately 70% of the brain’s energy is consumed to support intrinsic communication between neurons and non-neural cells (Raichle and Mintun 2006; Shulman et al. 2004; Tomasi et al. 2013). This intrinsic communication is generally observable as oscillating patterns of synchronized activity in distinct brain regions that can be measured with fMRI, allowing the identification of neural functional connectivity networks (Damoiseaux et al. 2006). Perhaps the best investigated of these networks is the “default mode network” (DMN), that mainly comprises the medial parietal, inferior parietal and ventromedial frontal cortex and is found to be strongly associated to the brain’s so-called resting state, i.e. to passive mind states and the absence of goal-directed activity (Raichle et al. 2001). The medial posterior parts of this network are metabolically very demanding, exhibiting the highest level of glucose consumption compared to any other region of the cortex in humans and other species (Gusnard et al. 2001; Vogt and Laureys 2005).

The dependence of normal brain function on a continuous glucose supply from the periphery via blood flow inevitably gives rise to questions about the interaction between changes in BGL and changes in regional cerebral blood flow (rCBF) as well as the blood-oxygen-level-dependent (BOLD) signal, which arises through the uncoupling of CBF and oxidative glucose metabolism during increased neuronal activity (Fox et al. 1988). Such questions have been addressed by numerous studies in the past mainly in the context of hypoglycemia both in healthy participants and diabetic patients. A series of studies have reported an attenuation of the BOLD signal in response to visual and auditory stimulation, motor activation and cognitive tasks during hypoglycemia (Anderson et al. 2006; Bolo et al. 2011; Driesen et al. 2007; Kennan et al. 2005; Rosenthal et al. 2001), whereas some studies showed region specific changes of CBF in response to hypoglycemia (Kennan et al. 2005; Teves et al. 2004). Insulin resistance in type 2 diabetes has been associated with reduced functional connectivity in the DMN (Musen et al. 2012), while one study found a persistent activity (decreased deactivation) of the DMN during a working memory task in hypoglycemia in patients with type 1 diabetes. This effect was most prominent in the posterior cingulate cortex (PCC) (Bolo et al. 2011). Interestingly, a recent study found that hyperglycaemia leads to a decreased deactivation of the DMN during a cognitive task in patients with type 2 diabetes (Marder et al. 2014). Despite the expanding knowledge on the effects of hypo- or hyperglycaemic blood glucose levels on brain function, potential effects of BGL at the low end of the normal range, i.e. BGL that can be encountered in healthy individuals several hours after their last meal, have not yet been systematically addressed. Previously, we showed that naturally occurring, low BGL do not have a strong systematic influence on the BOLD signal per se, yet this state interacted with attention related brain activation (Kohn et al. 2014). Nevertheless, influences of this state on large scale brain network dynamics have not been thoroughly investigated.

In the present study, we investigate the influences of naturally occurring, low BGL on intrinsic brain activity measured with fMRI. To this aim, we conducted a double-blind, placebo controlled study in which brain activity of fasting subjects was measured with fMRI at rest in two different conditions; once during administration of placebo, resulting in prolonged low BGL, and once during administration of glucagon, resulting in elevated BGL. We assumed that borderline low BGL could possibly lead to altered patterns of spontaneous brain activity, considering the relatively restricted glucose availability in the blood supplying the brain.

In the context of self-regulation, which can be conceptualized as a meta-theoretic approach for a broad range of cognitive, affective and social processes (Carver and Scheier 2011), an influential view on the phenomenon of ego depletion (Baumeister et al. 2007) proposes glucose as the central ‘limited resource’ for self-regulation (Gailliot and Baumeister 2007). While ego depletion and the limited resource model of self-regulation have both been challenged recently (Carter et al. 2015; Hagger and Chatzisarantis 2013, 2014; Inzlicht et al. 2015), we nevertheless wanted to investigate whether there is evidence for an influence of low-normal blood glucose on resting state networks that have been related to cognitive or attentional control processes, such as the executive control network and frontoparietal attention networks (Smith et al. 2009).

## Materials and methods

### Subjects

Out of 40 subjects in the initial study sample 30 (15 male) healthy, young (mean age 24,5 years, SD = 3,4), right-handed (Oldfield 1971) volunteers were included in this analysis. 10 subjects were excluded from the analysis for technical reasons concerning either the anatomical or functional resting-state scans (bad quality or missing data due to time restrictions). All subjects gave written informed consent and were monetarily compensated for their participation. Subjects were recruited through flyers posted on campus and screened on the phone for right-handedness and MRI suitability. Suitable participants underwent physical and neurological examination, blood tests for glucose, insulin, epinephrine and nor-epinephrine as well as neuropsychological testing and screening for mental disorders (SKID-PIT-light). Participants on any type of medication except oral contraceptives, with a history of metabolic, cardiovascular, neurological or psychiatric disorders, abuse of psychoactive substances, head trauma, allergies, BMI outside the range of 20–25 kg/m^2^, abnormal findings on examination, abnormal blood results or BGL > 80 mg/dl prior to fMRI measurement were excluded. The study was conducted in accordance with the Declaration of Helsinki in its 2008 revised form and was reviewed and approved by the ethical review board of the University Hospital RWTH Aachen.

### Design and procedure

Prior to fMRI measurements subjects had been fasting (i.e. not consuming food or any beverages that might contain carbohydrates, fat, protein, alcohol or artificial sweeteners) for at least 14 h. Participants were told to refrain from physical exercise on the days of the experiments because physical exercise can acutely improve insulin sensitivity and deplete glycogen stores of the liver. Measurements started between 12 am and 1 pm and lasted for approximately 60 min. FMRI measurements were conducted in two different conditions: one with no manipulation of glucose metabolism (FC, fasting condition), where subjects received an intravenous saline solution, and one where BGL were raised by administration of an intravenous infusion of glucagon hydrochloride (EC, elevated BGL condition). All interventions were conducted under double-blind conditions. Each subject underwent both fMRI measurements, resulting in a total number of 60 measurements. Half of the subjects were randomly assigned to FC for the first measurement and EC for the second and the other half vice versa. Time between the two fMRI sessions was on average 28 days.

Each fMRI session consisted of a resting state measurement followed by two major tasks in a counterbalanced order: a working memory paradigm and a mood induction paradigm (Schneider et al. 1994). These paradigms were followed by a basal visual stimulation (checker board). The fMRI session ended with a further resting state measurement and in one session an anatomical scan. Results concerning the main paradigms and the visual stimulation are reported elsewhere (Chechko et al. 2014; Kohn et al. 2014, 2015).

Resting state measurements were 6 min long. Subjects were asked to keep their eyes open while they were shown a grey screen (no fixation cross), let their mind wander and refrain from occupying themselves with anything specific.

Subjects in EC received an intravenous infusion of glucagon hydrochloride (GlucaGen® Hypokit, powder, Novo Nordisk, Mainz, in 50 ml Ampuwa ®, Fresenius Kabi, Bad Homburg) administered as a bolus injection of 1 mg glucagon hydrochloride prior to the beginning of the fMRI scan followed by continuous infusion of 0,5 mg/h during the session, resulting in a total administration of 1,5 mg glucagon. Subjects in FC received an intravenous NaCl 0,9% solution of the same total volume of 50 ml (NaCl, 0.9%, B. Braun, Melsungen). Glucagon is a polypeptidic hormone excreted by the α-cells of the pancreas. Its main function is the increase of hepatic glucose output through inhibition of glycogen synthesis and stimulation of both glycogenolysis and glyconeogenesis (Lefèbvre 1995), thereby glucagon administration mirrors a re-feeding situation after prolonged fasting. Infusions were administered through a peripheral venous catheter placed in the left upper limb using a perfusion pump (MRI-Caddy, Medfusion Inc., Duluth, Georgia, USA).

Intravenous blood samples were taken from a second peripheral venous catheter placed in the right upper limb 5 min after the bolus injection and at the end of each fMRI session to determine levels of epinephrine, nor-epinephrine, insulin, and cortisol. Blood glucose levels were measured before the bolus injection (t0), before each one of the two main paradigms and before visual stimulation (t1, t2, t3) as well as at the end of the session (t4) using capillary blood by finger stick via “Contour” blood glucose meter and test strips (Bayer Vital GmbH, Leverkusen, Germany) according to the manufacturer’s recommendations. With 99.3% accuracy, the Contour meter meets International Organization for Standardization accuracy requirements (ISO15197:2003).

Low euglycemic BGL were achieved naturally, i.e. they were accompanied by all hormonal and emotional responses occurring in a subject in a prolonged postabsorptive or fasting state, e.g. when being on a restrictive diet or simply having skipped breakfast. To raise low BGL we avoided oral carbohydrate administration as their taste triggers reward related brain activation (Chambers et al. 2009) and there are strong interindividual differences in resorption rates. We chose the parenteral administration of glucagon which is, in contrast to the glucose-clamp technic, applicable as a single infusion and demands no strict BGL-monitoring, thus enabling a double-blind design, while raising BGL sufficiently and promptly.

### Physiological and hormonal data analyses

Differences in BGL between the two groups were calculated using a paired t-test of mean BGL in FC and EC and a paired t-test of BGL before measurement. All analyses were tested for significance at a threshold of *p* = 0.05.

Epinephrine, norepinephrine and insulin levels were analyzed in comparison to pre-screening values in three separate repeated measures ANOVAs. Additionally, we analyzed cortisol values between EC and FC using a paired t-test.

### FMRI data acquisition and processing

Scanning was performed in the University Hospital RWTH Aachen. Imaging data was acquired in a 3T Trio Tim MRT (Siemens, Erlangen, Germany) using echo planar imaging (EPI), with TR = 2300 ms, TE = 30 ms, 38 slices, slice thickness = 3.5 mm. interslice gap = 0.35 mm, matrix size = 64 × 64, flip angle = 77°; field of view = 240 × 240 mm; 154 repetitions.

Resting state analysis was carried out in FSL (FMRIB’s Software Library, http://www.fmrib.ox.ac.uk/fsl; (Beckmann and Smith 2004)). Pre-processing steps included three-dimensional movement correction, brain extraction, and spatial smoothing using a 6 mm full-width at half maximum (FWHM) Gaussian kernel to reduce inter-subject variability and a high-pass filter (> 0.007 Hz) was applied. All pre-processing steps except temporal filtering were conducted before AROMA data denoising (Pruim et al. 2015a, b). Briefly, ICA-AROMA is designed to identify motion related artifacts by matching single subject ICA components to four robust and standardized features. The data is denoised by linear regression of ICA components identified as noise by AROMA and subsequently the high pass filter was applied. Additionally, individual white matter and CSF masks were calculated (by overlap between tissue prior maps and segmentation of the anatomical image) and both time-courses were regressed out of the functional data. Prior to all group analyses data were normalized to MNI space and re-sampled to 2 mm^3^ resolution using FMRIB’s Nonlinear Image Registration Tool (FNIRT).

For analyses of differential functional connectivity we selected four resting state networks of interest (RSNoI): (1) default mode network as we have previously shown that default mode network connectivity is differentially modulated during a mood regulation task in this sample (Kohn et al. 2015); (2) the executive control network was selected because of its association to cognitive control processing, which according to a prominent line of research on self-regulation may be detrimentally influenced by low-normal blood glucose levels (ego depletion;(Gailliot and Baumeister 2007)); (3) lateralized frontoparietal attention networks as those networks are also associated to cognitive processes related to the ego-depletion effect (Corbetta and Shulman 2002; Rottschy et al. 2012). Unthresholded z-maps of these RSN were taken from a canonical set of 10 well-mapped RSN (Smith et al. 2009) concatenated in a single 4D file and used as input for the dual-regression procedure (Filippini et al. 2009). The dual regression procedure was used to generate subject-wise maps of the four target RSN. In the first step, subject-specific temporal dynamics are identified by regression of the four target maps on each individual. The resulting time-courses for each RSNoI in each subject was next used to generate subject-wise spatial maps. To investigate condition differences, the resulting subject-wise RSN maps were subtracted according to the contrasts of interest. This resulted in subject-wise maps for the four components for EC versus FC (at R2) and R2 versus R1 (in FC and EC for control purposes). These subject-wise maps were then subjected to a permutation test for mean differences in dependant samples (Winkler et al. 2014). Correction for multiple comparisons within a volume is computed with threshold-free cluster enhancement (TFCE) (Smith and Nichols 2009) and correction for multiple comparisons regarding contrasts using the Bonferroni’s method.

## Results

### Analysis of blood glucose levels

**Fig. 1:**
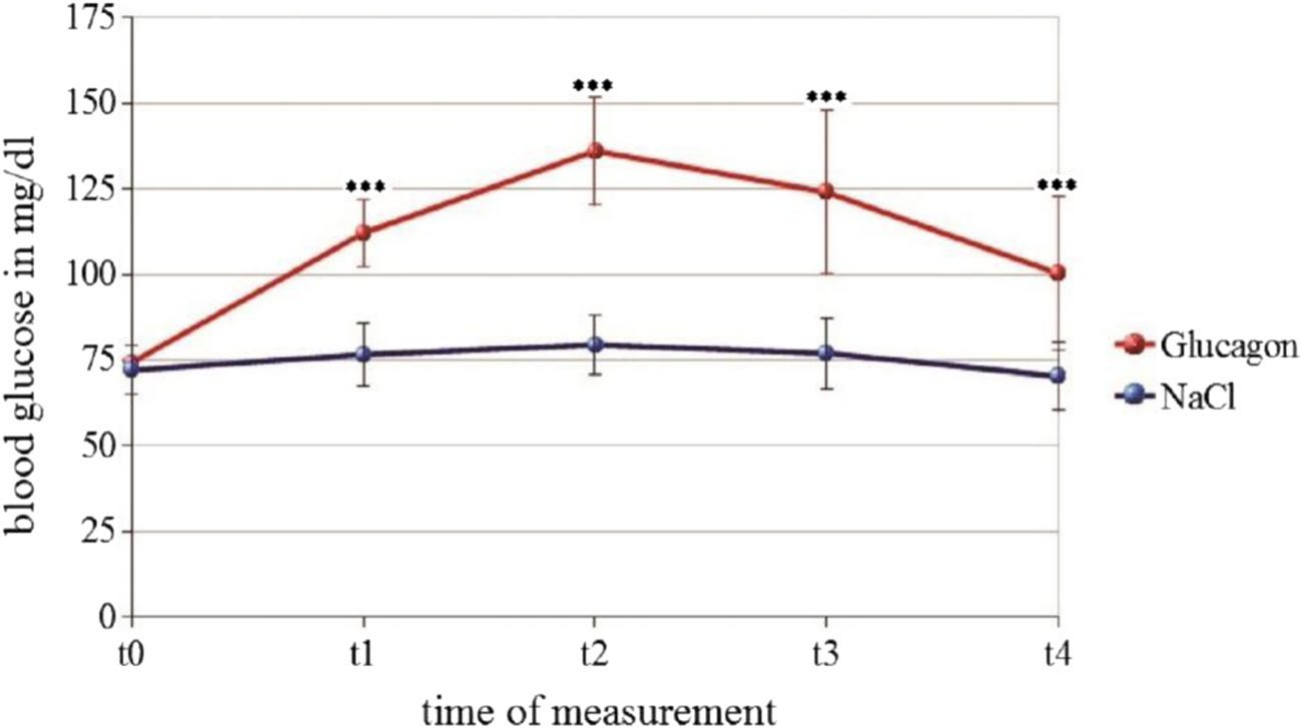
BGL under EC are displayed in red, BGLs in FC in blue. BGL were sampled approximately every 15 min. t0 refers to the time just before fMRI measurements began. t4 refers to the time just after fMRI measurements were completed. Blood glucose levels represent mean values. ***: *p* < 0.001. Graphic taken with permission from (Chechko et al. 2014)

BGL measured by finger prick from all participants prior to each fMRI session (t0) did not show significant differences between the FC and EC groups (mean EC = 72 mg/dl; mean FC = 71.2 mg/dl). During the experiment, FC and EC differed significantly at all further time points (t1 - t4) (mean EC = 114.5 mg/dl, mean FC = 73.9, t = 9.12, *p* < 0.05; Fig. 1). As reported previously, we observed a decrease in blood glucose in the FC condition over time, that is, blood glucose shows a significant decrease (after an initial minute increase) over the course of the experiment (Kohn et al. 2015). Following the experiment, subjects were asked whether they thought they had been administered glucagon or sodium chloride and answers lay below chance level.

### Hormonal analysis

Insulin levels differed significantly between FC and EC (FC insulin mean = 44.34 mU/l, SD = 23.2; EC insulin mean = 3.83 mU/l, SD = 1.7; *p* < 0.001). Adrenalin levels differed significantly between FC and EC (FC adrenalin mean = 179.64 pmol/l; EC adrenalin mean = 220.75 pmol/l; *p* < 0.045). There were no significant differences in noradrenalin levels. A more detailed presentation and a discussion of these results are reported elsewhere (Kohn et al. 2014).

### Resting state fMRI

**Fig. 2:**
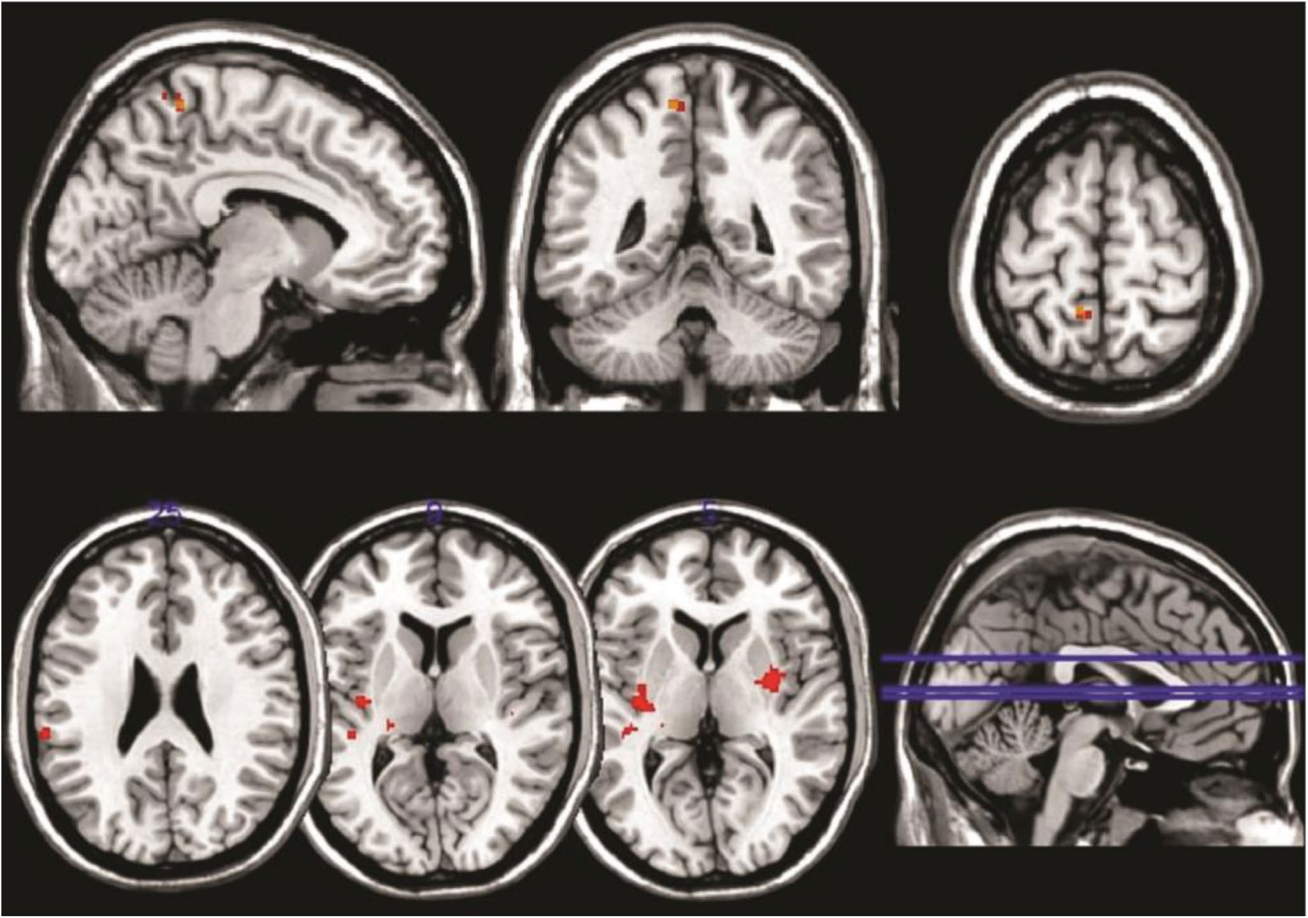
Differences between EC and FC in the DMN displayed in the upper part of the figure; differences between R1 and R2 in FC in the right frontoparietal network in the lower part of the figure. Results are superimposed on a standard brain (MNI 152). Areas shown were stronger connected to the rest of the network in EC compared to FC (upper part) and during R1 compared to R2 (lower part). Both contrasts are TFCE corrected at *p* < 0.05

After correcting for multiple comparisons, dual regression analysis performed for each of the RSNoI to search for differences between FC and EC during the second resting state measurement (EC versus FC on R2) showed significant differences only in the default mode network (DMN) (Fig. 2). Here, a small area in the left precuneus/superior parietal lobule exhibited greater connectivity with the rest of the network after administration of glucagon (EC) than after placebo (FC) (Table 1).

**Table 1:** Significant differences in functional connectivity for the four resting state networks of interest

Area	Hemisphere	Cluster extent	MNI coordinates			*t*-value
			x	y	z	
DMN; EC > FC						
Superior parietal lobule (SPL)	L	18	-8	-46	64	6.44
DMN; FC > EC						
No significant differences						
Right frontoparietal network; R1 minus R2 in FC						
Insula	R	84	34	-6	-4	3.83
Pallidum	R		20	-8	-2	3.43
Insula	L	66	-36	-16	6	4.07
Inferior parietal lobule	L	42	-60	-32	24	3.25
Parietal operculum	L	21	-42	-32	6	3.67
Superior temporal gyrus	L	19	-60	-28	4	3.47
Right frontoparietal network; R2 minus R1 in FC						
No significant differences						

Only the DMN and the right frontopariental network are listed as all other networks did not yield significant results. Clusters are listed with peak voxel location and respective local maxima (indented). For all peak voxel locations MNI coordinates and t-value are listed. Cluster extent is given in the peak voxel line only. Indented labels are local maxima of cluster above. All contrasts are TFCE-corrected at *p* < 0.05

*EC* elevated condition, *FC* fasting condition, *R1* first resting state scan at beginning of study, *R2* second resting state scan after 1 h of experimental tasks

Furthermore, dual regression analysis was performed in FC to reveal differences in functional connectivity between the two resting state sessions (R1 and R2). After correcting for multiple comparisons, such differences were found only in the right frontoparietal network, where an increased connectivity to the network was shown during R1 in the right posterior insula extending into the putamen and pallidum, left posterior insula, parietal operculum, superior temporal gyrus and inferior parietal lobule as well as in a series of smaller voxels in the right anterior thalamus, left posterior thalamus, left middle temporal gyrus and left cerebellum (Fig. 2; Table 1). No significant differences were found in the control analysis for R1 and R2 in EC.

## Discussion

In this placebo controlled, double-blind study we aimed to investigate the impact of naturally occurring, physiologically low blood glucose levels on intrinsic brain activity measured with fMRI. In the past, fMRI studies have addressed the relationship between the BOLD signal and BGL focusing on hypo- or hyperglycemia, conditions that are associated with endocrinological pathologies and do not occur in healthy individuals under normal circumstances. Furthermore, while several of these studies were able to replicate the finding of an attenuated BOLD signal in response to activation in hypoglycemia, the interpretation of their results in terms of BGL-induced changes in brain activity involves an important caveat: hypoglycemic BGL are accompanied by an increase in baseline CBF and, as previously shown, an increased baseline CBF can lead to a diminished BOLD signal due to a limited CBF response to subsequent neural activation (Paulson et al. 2010). On the contrary, BOLD signal changes measured during euglycemia are not confounded by hemodynamic changes and thus, can more reliably be interpreted as evidence of changes in neuronal activity.

In our study, we included fasting participants with BGL below 80 mg/dl to test the hypothesis that low euglycemic BGL could lead to changes in brain function due to the relatively diminished availability of glucose from the periphery to the central nervous system. Recently, such changes were reported during visual stimulation (Kohn et al. 2014), mood induction in relation to prior cognitive effort (Kohn et al. 2015) and cognitive performance (Chechko et al. 2014). Since passive mind states are hardly less energetically demanding than goal-directed activity (Raichle and Mintun 2006; Sokoloff et al. 1955), one could expect low BGL to also affect functional brain connectivity during rest. Indeed, model free analysis of our data revealed enhanced connectivity of a small cluster within the posterior DMN after glucose levels were elevated (EC) as well as a disruption of functional connectivity in the right frontoparietal network after cognitive effort (series of paradigms) in the fasting state (FC). Importantly, no changes were found in two other major resting state networks: the left frontoparietal and the executive control network.

### Changes in the default mode network

Since its first identification 15 years ago (Raichle et al. 2001) the DMN has triggered an exponentially increasing number of publications (Callard and Margulies 2014), leading among others to a large body of evidence that demonstrates the susceptibility of the network’s coherence to a series of conditions and manipulations. Research so far suggests that the DMN is disrupted during conscious sedation (Greicius et al. 2008), deep sleep (Horovitz et al. 2009), through disorders of consciousness (Vanhaudenhuyse et al. 2010), sleep deprivation (Gujar et al. 2010) and normal aging (Mevel et al. 2013) as well as in many neuropsychiatric diseases (Fox and Greicius 2010; Zhang and Raichle 2010). Here, we report findings that for the first time indicate changes within the DMN after overnight fasting. Fasting of approximately 15 h resulted in BGL below 80 mg/dl and diminished functional connectivity of a small midline area within the posterior DMN compared to a state of elevated glucose levels after glucagon infusion.

Recent studies combining fMRI with fluorodeoxyglucose-PET (FDG-PET) have demonstrated a positive correlation between local glucose metabolism and the degree of functional connectivity in the DMN (Aiello et al. 2015; Passow et al. 2015; Tomasi et al. 2013). Since the precuneus is one of the most metabolically active brain regions (a so-called “hot spot”) (Cavanna and Trimble 2006), the maintenance of its metabolic rate and thus, the degree of its functional connectivity, when glucose supplies are limited may not be possible, leading to some degree of disconnection from the rest of the network under diminishing BGL. However, as our results indicate, the impact of physiologically low BGL on intrinsic brain activity appears to be limited: after approx. 15 h of fasting only a small cluster within the DMN was affected while three other major intrinsic networks showed no significant changes.

### Changes in the right frontoparietal network

Prior to the second resting state measurement, all subjects had participated in a mood induction and a working memory task as well as a session of visual stimulation. Comparison of functional connectivity during the initial (R1) and final (R2) resting state scan under continuously low BGL revealed a disrupted connectivity in the right frontoparietal network in R2. Changes were more prominent in the posterior putamen and posterior insula bilaterally, but there were also significant changes in smaller cortical and subcortical regions, mainly in the left hemisphere as well as in the left cerebellum.

As we observed significant decreases in BGL over the course of the whole experiment in FC only, functional connectivity differences could be related to decreased BGL and this would be in line with the strength model of self-regulation relying on BGL (Gailliot and Baumeister 2007). Nevertheless, as these differences are only observed in one frontoparietal network our results should be interpreted with caution and could very well also relate to interaction of low BGL and previous attentional load.

Both frontoparietal networks (FPN) are associated with attention, cognition and language, whereas the right FPN is particularly associated with perception, somesthesis and pain (Smith et al. 2009). A diminished involvement of the posterior insula in the right FPN at the end of our experiment in FC may be indicative of a connection between BGL and nociception (Segerdahl et al. 2015), although further studies including behavioral data would have to substantiate this assumption.

## Limitations

Although glucagon administration was proven to raise BGL effectively and be suitable for a double-blind design, it does theoretically constitute a potential confounding factor influencing spontaneous BOLD fluctuations and study results. Glucagon is found to enter the blood brain barrier and bind at multiple receptor sites in olfactory tubercle, hippocampus, anterior pituitary, amygdala, septum, medulla, thalamus, olfactory bulb and hypothalamus (Authier and Desbuquois 2008). Central glucagon action is found to influence neuronal activity leading to appetite suppression, presumably by hypothalamic corticotropin-releasing factor stimulation and thus triggering of the hypothalamic–pituitary–adrenal axis (Filippi et al. 2013; “Glucagon - FDA prescribing information, side effects and uses,” n.d.). On the other hand, glucagon has been found to have no influence on cortical neurons (Inokuchi et al. 1986). As we did not measure CNS-glucagon concentrations, any discussion about glucagon-induced changes on patterns of neuronal activity has to remain speculative. We would argue though, that the to date known central actions of glucagon, although of potentially confounding nature seem insufficient to exclusively account for the changes in intrinsic brain activity found in our study, as these affected the cortex and part of a well-defined neural network.

Finaly, although we find glucagon administration to be the best way of elevating BGL in a double-blind experiment, one should only carefully attempt to apply conclusions based on our data to neuronal processes in the postprandial state, as previous reports have shown that endocrinal and neural signalling originating from the gastrointestinal tract evokes different brain signalling patterns than manipulations of the metabolic state induced by parenteral interventions (Smeets et al. 2007).

## Conclusion

Overall, our study indicates that overnight fasting has a limited impact on intrinsic functional connectivity of the healthy human brain. From four major resting-state networks tested, fasting appeared to affect network coherence only in a small area within the posterior default mode network. Furthermore, we found evidence that at low BGL functional connectivity can become disrupted after cognitive effort, suggesting a diminished capacity of the “fasting” brain to maintain its activity when there is external demand. These results are largely in line with the limited resource model of self-regulation and its reliance on glucose (Baumeister et al. 2007; Gailliot and Baumeister 2007), although it has to be noted, that only one of three self-regulation related resting state networks showed a significant difference, which was not located in putative major hubs related to cognitive control and self-regulation (Heatherton and Wagner 2011). Thus, we would argue that if ego depletion mediates the influence of BGL on functional connectivity, such mediation would have to be indirect.

The aim of this study was to search for changes in the BOLD signal caused by fluctuations of blood sugar levels in healthy subjects. Longer fasting periods or greater increases in BGL than the ones induced in our experiment (e.g. postprandial) may lead to greater changes in intrinsic brain activity than the ones reported here.
